# On Rheumatism, Rheumatic Gout, and Sciatica, Their Pathology, Symptoms, and Treatment

**Published:** 1854-08

**Authors:** 


ART. V.—*On Rheumatism, Rheumatic Gout, and Sciatica, their pathology, symptoms, and treatment.* By HENRY WILLIAM FULLER, M. D., Cantab., Fellow of the Royal College of Physicians, London; Assistant Physician to St. George's Hospital, &c., &c. New York: Samuel S. & William Wood. 1854.

A volume of three hundred and twenty pages is here devoted to a special disease. To all who have felt that rheumatism, especially in its chronic forms, is one of the most unsatisfactory diseases, as to treatment, which come under the cognizance of the physician, this volume recommends itself by its scientific merit, no less than by a pure and readable style of scientific composition.

It is a pleasure to recommend this work to our readers. The space given to its consideration enables the author to develop all the features of the disease, and give to each a careful notice. The frequency of rheumatic attacks within the temperate zone, the regular laws by which it is governed, and the obstinate character of its symptoms, render it a subject deserving of study. The author, in the introduction, considers the causation of the disease to be a special poison, viz: an excess of lactic acid in the blood. All other causes, as exposure to cold and wet, are ranked as exciting causes only; and not productive of rheumatism, except as they may hasten the development, or retard the elimination of lactic acid. The argument by which this position is reached, is an able, and to our mind a conclusive one. The law of parallelism, as shown in a recent article in this journal, and its frequent metastatic changes, are sufficient to separate it from the ordinary phenomena produced by cold or other common causes.

In speaking of parallelism, the author allude, scursorily, to the explanation of this singular phenomenon offered by Mr. Paget, in his recent valuable work on Surgical Pathology. Mr. Paget supposes that nutrition and removal of effete matter go on symmetrically in parallel organs, and that consequently both ancles, or both elbows, will be in the same stage of growth and repair at any given time; and so afford a tissue on either side of the body equally open to the attacks of disease. The theory is at least a plausible, and very probably the true explanation.

In a subsequent chapter we have a statistical proof of the hereditary character of rheumatism, with a table exhibiting the ages most liable to its attack:

Of 289 patients having acute rheumatism,

| | | |
|---|---|---|
| 16 | were between the ages of | 5 and 15; |
| 241 | " " " | 15 " 40; |
| 25 | " " " | 40 " 50; |
| 7 | had passed the age of 50. | |

It seems that age and constitution have an effect in modifying the acuteness of the attack. The infirm, like the aged, may have sub-acute, or chronic rheumatism; while the vigorous old, may, like the robust young, be subjected to acute attacks.

On the subject of treatment, we quote from page 72, the following judicious remarks:

"The bleeding, which in the young, plethoric, and robust, may be necessary to allay excessive vascular action and cause free secretion, may, in the weakly, induce irritability of the heart, and a consequent attack of cardiac inflammation. The opium, which in one person may prove of the greatest service in promoting free perspiration, and in allaying the general irritability of the system, may, in another, check the biliary and other secretions, and thus prevent the elimination of the rheumatic poison. The continued use of calomel, and the constant purging, which may be beneficial to one patient by removing large quantities of unhealthy secretions, may unnecessarily exhaust the strength of another, and tend, very greatly, to impede recovery. And so in regard to every remedy which has been proposed that is useful at one time, proves useless or positively injurious at another; and the conclusion is forced upon us, that what is wanted, "is far less the discovery of untried methods of treating disease, than of discriminative canons for the proper use of those we possess;" far less the discovery of any new medicines, than the adaptation of our present remedies to the exigencies of each case."

Dr. Fuller then takes up the various remedies which have been relied upon in rheumatism, and examines the fitness of each for the purpose designed. Blood-letting, when freely used, is spoken of as increasing the liability to heart complication, and as likely to leave the patient in a feeble and sickly condition. Even in cases of a sthenic form, accompanied by much fever, it is asserted that, as there is very little tendency to dangerous results, bleeding is usually unnecessary. Some cases—those of the young with plethora, high fever, and dryness of skin—may be benefited by slight venesection.

The action of calomel and purgatives is more favorably mentioned. They rid the intestinal canal of the offensive secretions often present; by stimulating a large surface to secretion, they remove much fluid from the system; and, of course, a portion of the rheumatic poison. Frequent and repeated purgings are, however, objected to as increasing the irritability of the system, and enfeebling it without adequate compensation in benefits derived.

We have not space to follow our author through, and although the book is a somewhat lengthy one, we find that it is not easy to convey its ideas in terms more concise than its own. We can only express our general concurrence in its doctrines, and recommend it to our readers as a truly valuable and instructive treatise. It is one of a class of works based on individual research, and study into special subjects, which are fast taking the precedence of works on general practice; and substituting definite and thoroughly digested facts, for the crude theories of mere students.

Our readers will also notice that the humoral pathology pervades and gives life and form to the doctrines of the author. This is another of the signs of the times, and is indicative of the rapid returning of the medical mind to this most simple, obvious, and truthful theory of disease.

For sale by Chas. J. Leonard. H.

